# Clinical and Paraclinical Screening for Celiac Disease in Children with Intractable Epilepsy

**DOI:** 10.1155/2021/1639745

**Published:** 2021-04-22

**Authors:** Golnaz Ghazizadeh Esslami, Bahar Allahverdi, Reza Shervin Badv, Morteza Heidari, Nahid Khosroshahi, Hosein Shabani-Mirzaee, Kambiz Eftekhari

**Affiliations:** ^1^Department of Pediatrics, Ziaeian Hospital, Tehran University of Medical Sciences, Tehran, Iran; ^2^Pediatric Gastroenterology and Hepatology Research Center, Children's Medical Center, The Pediatric Center of Excellence, Tehran University of Medical Sciences, Tehran, Iran; ^3^Department of Pediatric Neurology, Children's Medical Center, Pediatrics Center of Excellence, Tehran University of Medical Sciences, Tehran, Iran; ^4^Department of Pediatric Neurology, Children's Medical Center, Pediatrics Center of Excellence, Department of Pediatric Neurology, Vali-e-Asr Hospital, Imam Khomeini Hospital Complex, Tehran University of Medical Sciences, Tehran, Iran; ^5^Department of Pediatrics, Bahrami Children's Hospital, Tehran University of Medical Sciences, Tehran, Iran; ^6^Pediatric Gastroenterology and Hepatology Research Center, Tehran University of Medical Sciences, Department of Pediatric, Bahrami Children's Hospital, Tehran, Iran

## Abstract

**Background:**

Celiac disease is the inflammatory entropy caused by hypersensitivity to gluten, which occurs in susceptible individuals. Some studies have suggested a link between celiac disease and epilepsy in children. Our aim was to screen for clinical and paraclinical features of celiac disease in children with intractable epilepsy.

**Methods:**

This was a cross-sectional study. Children aged 2 to 18 years with refractory epilepsy that referred to the pediatric neurology clinic within one year (2018–2019) were enrolled. Demographic and clinical characteristics of patients, especially clinical manifestations of celiac disease, were recorded in a questionnaire. A venous blood sample was sent to determine the total IgA, anti-tTG (IgA), and anti-endomysial antibody (IgA). Endoscopy was performed in cases where the celiac serological test was positive.

**Results:**

Seventy children with idiopathic drug-resistant epilepsy (44 boys) were evaluated. The height-for-age index was 49.2% and the weight-for-age index was 38.2% less than normal. Constipation (48.6%), anorexia (25.7%), and abdominal pain (21.4%) were the most common gastrointestinal symptoms. Celiac serological tests were negative in all children. Therefore, endoscopy and bowel biopsy were not performed in any case.

**Conclusion:**

Celiac disease was not found in any patient with intractable epilepsy. Gastrointestinal symptoms and growth disorders in this group may be related to the underlying disease or medications and not to celiac disease.

## 1. Introduction

Celiac disease (CD) is a chronic autoimmune disease that affects the small intestine [1]. The disease is caused by a reaction to gluten in those who are genetically predisposed [1]. CD is classified into different types: obvious, silent, and latent [1]. The prevalence of the obvious type of disease is reported to be 1% in the general population [1]. The most common clinical manifestations of the disease in children are gastrointestinal problems, which are characterized by diarrhea, constipation, abdominal distention, malabsorption, loss of appetite, growth retardation, and short stature [1]. CD may initially present with neurological manifestations; seizures and epilepsy with bilateral occipital calcifications could be one of the clinical manifestations of the silent disease [1, 2]. Some reports have suggested a link between celiac disease and neuropathy, ataxia, seizures, and dementia. The possibility of a connection between celiac disease and epilepsy in adolescence is discussed in some articles [3–8]. Therefore, screening of CD may be necessary in patients with the neurological disorders [9]. The screening panel of CD is usually based on serological testing especially tissue transglutaminase antibody (tTG IgA) (with high sensitivity and specificity) and anti-endomysial antibody (EMA) (to confirm the tTG IgA result), and the definitive diagnosis is made by pathological findings on the small bowel biopsy [2]. Our aim was to screen for celiac disease in terms of clinical manifestations and paraclinical findings in children with intractable epilepsy. Intractable epilepsy is defined as seizures attacks that are not controlled with two appropriate anticonvulsant medications. If CD was proven and treated by a gluten-free diet, seizure control could be better in patients with intractable epilepsy.

## 2. Materials and Methods

This was a cross-sectional study performed over one year (2018–2019). Children aged 2–18 years with intractable seizures referred from the Children's Neurology Clinic of Children's Medical Center and Bahrami Children Hospital were enrolled in the study.

Exclusion criteria were children with celiac disease, history of corticosteroids consumption, ketogenic regimen, and/or history of antiepileptic surgery and children with an identified cause for epilepsy such as metabolic diseases, brain trauma, and encephalopathy.

### 2.1. Sampling Method and Sample Size

The sample size included all 2- to 18-year-old children with intractable epilepsy referred from the Children's Neurology Clinic during one year, compliant with the study.

### 2.2. Intervention

Initially, written consent was obtained from all eligible children or their parents. Then, the questionnaire was completed including demographic and clinical characteristics such as age, gender, height, weight, age of onset of seizures, family history of epilepsy and celiac disease, names of antiepileptic drugs, and clinical symptoms of celiac disease (abdominal pain, vomiting, anorexia, diarrhea, constipation, clubbing, gluteal muscle atrophy, and short stature). The *Z*-score for height-for-age indicators (*Z*_H/A_), weight-for-age (*Z*_W/A_), and BMI-for-age (*Z*_BMI/A_) was calculated using the World Health Organization (WHO Anthro) software.

Five milliliters of intravenous blood samples was taken simultaneously with periodic sampling (liver enzymes and anticonvulsant drug levels) and sent to the Children's Medical Center laboratory. Tissue transglutaminase antibody levels (IgA) (anti-tTG IgA) were measured by enzyme-linked immunosorbent assay (ELISA) using Aeskulisa kit (Aesku.lab Diagnostics, Wendelsheim, Germany) and anti-endomysial antibody (IgA) (EMA IgA) by indirect immunofluorescence technique. Anti-tTG antibody levels ≥20 U/ml were considered positive (seropositive). The results of the anti-EMA IgA were reported to be positive or negative (seropositive vs. seronegative). Total immunoglobulin A (IgA) levels were measured by nephelometry method by using laboratory kit (MININEPHTM Human IgA Kit, The Binding Site Ltd., Birmingham, UK). The IgA levels less than 33 mg/dl were considered abnormal. The results of these tests were collected and recorded in the relevant questionnaires. Under 18 months, anti-tTG has little diagnostic value, so children older than two years were enrolled in our study. Children who had a positive serological test underwent endoscopy and a small bowel biopsy to confirm the disease.

### 2.3. Statistical Analysis

The data were analyzed using SPSS software version 25. The mean and standard deviation were used to describe the quantitative variables, and the number (percentage of frequency) was used to describe the qualitative variables. The chi-square test was used to analyze the data (the relationship between antiepileptic drugs and gastrointestinal symptoms). *P* value less than 0.05 was statistically significant.

## 3. Results

In this study, seventy children with intractable epilepsy were referred from a neurology clinic. Forty-four (62.9%) were boys. Their mean age was 7.8 ± 4.8 years. The mean age of patients at onset of epilepsy was 2.6 ± 3.1 years. The onset of seizures in most cases was less than 6 years. None of the patients had a gluten-free diet. Three patients suffered from hypothyroidism and one from diabetes mellitus type 1. Others were not affected by any other comorbidities or autoimmune diseases. Twenty-five patients (36.2%) had a family history of epilepsy. The family history of celiac disease was not found in any of the patients. The mean height (cm), weight (kg), and body mass index of the patients were 118.1 ± 28.9, 26.9 ± 18.5, and 16.9 ± 4.1, respectively. The mean *Z*_H/A_ in patients was −1.1 ± 1.7. The height of 27 children (42.9%) was within the normal range. The mean *Z*_W/A_ in patients was −0.8 ± 1.7. The weight of 32 children (47.1%) was within the normal range. The mean Z_BMI/A_ in patients was −0.7 ± 2.24. The BMI of 27 children (38.6%) was within the normal range (Table 1).

On average, each patient with intractable epilepsy had used about 4 (3–5) antiepileptic medications. The most commonly prescribed antiepileptic medications were sodium valproate (61.4%, *n* = 43), levetiracetam (58.6%, *n* = 41), phenobarbital (48.6%, *n* = 34), and carbamazepine (32.9%, *n* = 23), respectively. Constipation was reported in 34 (48.6%) and anorexia in 18 (25.7%). Abdominal pain was observed in 15 patients (21.4%), abdominal distension in 10 (14.3%), vomiting in 2 (2.9%), and diarrhea in one (1.4%). There was no case of finger clubbing or gluteal muscle atrophy. Total IgA serum was normal in all patients except one. In this case, anti-tTG IgG was used for celiac disease screening, in which it was normal. No abnormal anti-tTG (IgA) and anti-EMA (IgA) were reported. Endoscopy and intestinal biopsy were not performed, due to the negative serological tests. The association of four common antiepileptic drugs with three common gastrointestinal symptoms (constipation, anorexia, and abdominal pain) is summarized in Table 2.

## 4. Discussion

In recent years, special attention has been paid to the link between celiac disease and neurological diseases such as epilepsy. The autoimmune mechanism, generalized absorption disorder, and gluten-induced toxicity have been suggested to justify association between celiac disease and neurological diseases [10–12]. In our study, no children with intractable epilepsy had a positive serology for celiac disease. The prevalence of celiac disease in children with epilepsy is reported to be less than 1% to about 10% [10, 13, 14]. In the Pratesi study, there was no significant difference in the prevalence of celiac disease among patients with epilepsy and the control group [15]. Emami showed that, in children with epilepsy, anti-tTG (IgA) was positive in 3.7% and pathologically confirmed celiac disease in 2.8% [16]. In Dalgic's study, anti-tTG (IgA) was positive in 4.7% of patients [17]. In order to justify the difference in prevalence, we can point to differences in geographical area, race, and demographic characteristics of patients and also difference in study methods. In most previous studies, the groups consisted of children with uncomplicated idiopathic epilepsy, but our patients had idiopathic intractable epilepsy. Karimzadeh showed that positive serology of celiac disease in children with intractable epilepsy and uncomplicated idiopathic epilepsy was 6.8% and 2.4%, respectively [8]. Bruni founded that, after starting a gluten-free diet in a child with intractable epilepsy, recovery occurred and the EEG became normal. This child was 2 years old and had only positive HLA-DQ8, but no clinical symptoms of celiac disease or positive serology. This paper noted the potential effectiveness of a gluten-free diet (despite celiac disease) in the treatment of intractable epilepsy [18]. There were growth disorders (including height, weight, and BMI) in our patients. This finding is consistent with many previous studies, stating that, in children with epilepsy, the prevalence of growth disorders is high [13]. Our results, like previous studies, showed that *Z*_H/A_ was lower in these children [14]. Several factors play a role in the growth disorders in patients with epilepsy. For instance, multiple prolonged seizures, long postictal state, and short waking hours can reduce a patient's nutrition. On the other hand, the use of antiepileptic drugs also reduces appetite and induces vitamin deficiency especially vitamin D, which itself plays an important role in bone growth [19, 20]. There was no evidence of celiac disease in our patients, so their growth disorders were mostly due to underlying disease. The most common gastrointestinal symptoms in our patients were abdominal pain, anorexia, constipation, and abdominal distention. On the other hand, antiepileptic drugs can cause a number of gastrointestinal side effects such as nausea, vomiting, constipation, diarrhea, and dysphagia [21]. As a result, these gastrointestinal manifestations may be iatrogenic. In our study, there was statistically significant association between phenobarbital and anorexia; in other cases, there was no connection between medication and gastrointestinal manifestations. Emami found that abdominal pain and constipation were common in patients with epilepsy in the absence of celiac disease. However, diarrhea was significantly higher in celiac patients than in nonceliac patients [16]. Vieira showed that the most common gastrointestinal manifestations in patients with epilepsy were abdominal pain, constipation, abdominal distension, and diarrhea, respectively [22].

## 5. Conclusion

We examined the clinical symptoms and screening results of celiac disease in children with epilepsy. We did not find any case with positive serology of celiac disease. Abdominal pain, anorexia, constipation, and abdominal distension were the most common gastrointestinal symptoms in these patients. These symptoms were related to the underlying disease and were not related to celiac disease or medication.

### 5.1. Limitations of the Study

Lack of control group and low sample size were among the most important limitations of our study.

### 5.2. Suggestions

1. It is recommended to conduct future studies with a larger sample size and more additional laboratory studies and compare the results with the healthy control group
2. It is recommended that the effectiveness of a gluten-free diet in improving control of epilepsy in children with intractable epilepsy be evaluated.

## Figures and Tables

**Table 1:** The mean *Z*_H/A_, *Z*_W/A_, and Z_BMI/A_ in patients.

Z-score range	−2 <Z-score < −1	−2 <Z-score < −3	Z-score < −3
*Z*_H/A_	16 (25.4%)	7 (11.1%)	8 (12.7%)
*Z*_W/A_	8 (11.8%)	11 (16.2%)	7 (10.3%)
Z_BMI/A_	7 (10.0%)	3 (4.3%)	11 (15.7%)

*Z*_H/A_: *Z*-score height/age; *Z*_W/A_: *Z*-score weight/age; Z_BMI/A_: *Z*-score body mass index/age.

**Table 2:** The relationship between antiepileptic drugs and gastrointestinal symptoms in patients with intractable epilepsy.

Sodium valproate		Levetiracetam		Phenobarbital		Carbamazepine	
Variables	*N* (%)	*P* value	*N* (%)	*P* value	*N* (%)	*P* value	*N* (%)	*P* value
Constipation	19 (44.2%)	0.462	22 (53.7%)	0.341	21 (61.8%)	0.341	9 (39.1%)	0.315
Abdominal pain	11 (25.6%)	0.376	6 (14.6%)	0.140	10 (29.4%)	0.149	6 (26.1%)	0.545
Anorexia	12 (27.9%)	0.780	11 (26.8%)	1.00	13 (38.2%)	**0.028**	3 (13.0%)	0.145
Total	43 (100%)		41 (100%)		34 (100%)		23 (100%)	

## Data Availability

The data used to support the findings of this study are available from the corresponding author upon request.
